# Living Labs as catalysts for experiential learning in law enforcement training: Insights from the TENACITy project.

**DOI:** 10.12688/openreseurope.21573.1

**Published:** 2025-12-05

**Authors:** Konstantinos Margaros, Christiana Aposkiti

**Affiliations:** 1 Center for Security Studies (KEMEA), Athens, 10177, Greece

**Keywords:** Living Labs; experiential learning; andragogy; transformative learning; adult education; communities of practice; law enforcement training; TENACITy.

## Abstract

**Background:**

Living Labs have become established in European research as environments for user-centered innovation and co-creation. Yet, their potential as pedagogical infrastructures remains underexplored. This study investigates how Living Labs can foster experiential learning within EU-funded security research, focusing on the Horizon Europe project *TENACITy*, which integrates Living Labs into law enforcement training, offering a unique opportunity to examine how such settings can enhance adult and professional learning.

**Methods:**

A qualitative research design was applied, combining semi-structured interviews with officers from Passenger Information Units (PIUs) who participated in Living Lab sessions and document analysis of project materials, including Deliverable D4.1 *Training Methodology and Curricula*. Thematic analysis was used to identify patterns in participants’ accounts, interpreted through the theoretical lenses of experiential learning, andragogy, transformative learning and communities of practice.

**Results:**

Five interrelated dimensions of learning were identified: (1) active engagement and concrete experience, (2) processing and dialoguing, (3) linking experience to theory and practice, (4) testing and applying new knowledge and (5) preparation, support, and learning conditions. These dimensions demonstrate how Living Labs operationalize Kolb’s experiential learning cycle, embody Knowles’ principles of self-directed and problem-centered learning, align with Illeris’ holistic model integrating cognitive, emotional, and social dimensions, and reflect Wenger’s emphasis on collective meaning-making. Limitations included tool immaturity, reliance on synthetic datasets, and uneven facilitation.

**Conclusions:**

Living Labs represent hybrid ecosystems that combine innovation and learning. When intentionally designed and pedagogically facilitated, they can strengthen professional capacity building by linking technological development with authentic, reflective, and collaborative learning. The findings suggest that Living Labs should be embedded as integral, sustained components of training in EU-funded research projects, reinforcing the reciprocal relationship between innovation and education.

## Plain Language Summary

This article examines how Living Labs (collaborative environments where individuals test new tools and ideas in real-world settings) can also be used for professional learning. The study focused on the experiences of Passenger Information Unit officers who participated in Living Lab activities as part of the Horizon Europe project TENACITy, which aimed to develop data-driven tools to enhance border security and risk assessment. Through interviews with the participating officers, the study revealed that Living Labs are beneficial both for testing technology and for facilitating professional learning among colleagues. Officers indicated that they learned most effectively when sessions were well-organized, included real-life examples and provided opportunities for reflection and dialogue with peers. The research demonstrates that such environments encourage participants to connect theory with practice, experiment in a safe space and share knowledge within a community. The findings suggest that future European projects could use Living Labs more intentionally as training spaces, ensuring that innovation and professional education go hand in hand.

## Introduction

Living Labs have become an established approach in European research and innovation, valued for their capacity to support user-centered design, multi-actor collaboration, and experimentation in real-life settings ^1– 3^. Much of this literature has focused on their role in technological development and open innovation processes, emphasizing dimensions such as networks of actors ^4^, roles and participation ^5, 6^, and the principles and characteristics that define their outcomes ^7– 9^. While this work provides a rich understanding of how Living Labs function as innovation ecosystems, their potential as pedagogical infrastructures has been far less explored.

This article addresses this gap by examining how Living Labs contribute to adult learning in the context of EU-funded security research. European programmes such as Horizon Europe increasingly emphasize the training dimension of research projects, requiring that technological innovation be accompanied by capacity building among end-users. The TENACITy project (Grant Agreement 101074048) provides a pertinent case, as its Living Lab sessions were designed not only to test data-driven tools for Passenger Information Units (PIUs) but also to develop professional competences among participating officers. This dual purpose illustrates the emerging recognition that Living Labs can function simultaneously as innovation environments and as learning environments.

To analyze this pedagogical potential, the article draws on theories of experiential, adult, and social learning. Kolb’s ^10, 11^ experiential learning cycle highlights how knowledge is created through cycles of experience, reflection, conceptualization, and experimentation. Knowles’ ^12, 13^ andragogy emphasizes adults’ preference for self-directed, problem-centered, and immediately applicable learning. Mezirow’s ^14^ transformative learning theory stresses the role of critical reflection and dialogue in reshaping perspectives, while Wenger’s ^15^ concept of communities of practice highlights the collective and participatory nature of learning. Together, these frameworks provide a lens through which the pedagogical dimensions of Living Labs can be understood.

The study pursues two interconnected aims: first, to explore how participants experienced the pedagogical features of Living Labs in the TENACITy project; and second, to assess how these findings can inform the design of training components within EU-funded research. In doing so, the article contributes to both adult education scholarship and the practical development of professional training models for law enforcement.

## Literature review

### Living labs: conceptual foundations

Living Labs have emerged over the past two decades as a prominent framework for user-centered innovation, particularly within the European context. While the term is widely used, scholars emphasize different dimensions, reflecting the diversity of approaches to defining and operationalizing the concept. Følstad ^1^ describes Living Labs as environments for innovation and development carried out in real-life settings, stressing authenticity and direct user involvement. Bergvall-Kåreborn *et al.* ^2^ similarly frame them as “milieus” where various actors collaborate in open innovation processes, integrating technical, social, and organizational perspectives.

A complementary line of research highlights the networked nature of Living Labs. Leminen and Westerlund ^4, 5^ conceptualize them as networks of actors whose collaboration determines the direction and outcomes of innovation. This network orientation is further developed through typologies of roles ^5^ and participation modes ^6^, which underline the flexible and heterogeneous character of Living Labs. Schuurman *et al.* ^3, 16^ contribute by framing them as research approaches that combine co-creation with systematic methods of evaluation, thereby positioning Living Labs as both practice and methodology. Veeckman *et al.* ^8^ and Ståhlbröst ^7^ complement these perspectives by examining characteristics and principles that can be used to assess the effectiveness and impact of Living Labs.

Despite their definitional plurality, common features recur across the literature: user participation, multi-stakeholder collaboration, real-life experimentation, and iterative feedback processes. Westerlund and Leminen ^9^ argue that these features make Living Labs particularly well suited to environments where technological and social innovation must advance in parallel. Taken together, these studies point to a shared understanding of Living Labs as hybrid environments, simultaneously serving as contexts, processes, and networks where innovation and learning co-evolve.

### Open innovation and co-creation

The conceptual foundations of Living Labs are closely linked to the broader paradigm of open innovation. Chesbrough ^17^ introduced the idea that organizations should not rely exclusively on internal research and development but instead draw on external knowledge flows and collaborative processes. Living Labs embody this principle by creating structured settings where diverse stakeholders jointly shape and test emerging solutions.

The co-creation perspective is central to this approach. Leminen and Westerlund ^4^ emphasize that Living Labs function as arenas where companies, public bodies, users, and researchers collaborate on equal footing, each contributing complementary expertise. This resonates with the view of Bajmócy and Pataki ^18^, who connect co-creation to the framework of responsible research and innovation, highlighting that innovation should not only be effective but also inclusive and socially accountable. From this perspective, Living Labs provide a mechanism to ensure that innovation processes integrate ethical reflection and societal needs.

More recent work further underscores the role of Living Labs in education and community development. Ferrara *et al.* ^19^ illustrate how Living Labs can be employed to promote collaborative learning in local contexts, demonstrating their capacity to function simultaneously as sites of innovation and as educational environments. This broadens their relevance beyond the technological domain, positioning them as catalysts for capacity building and knowledge sharing.

To summarize, the literature on open innovation and co-creation highlights Living Labs as practical examples of collaborative innovation theory. They combine flexible organizational boundaries with structured engagement among various stakeholders, creating an environment that promotes both technological development and learning opportunities for professionals and community members.

### Experiential learning

Experiential learning provides a theoretical lens through which the pedagogical potential of Living Labs can be understood. Kolb’s ^10, 11^ model remains the most influential, conceptualizing learning as a cyclical process that moves through concrete experience, reflective observation, abstract conceptualization, and active experimentation. In this framework, knowledge is not transmitted but constructed through the transformation of experience, a process that aligns naturally with the real-life, participatory character of Living Labs.

Building on Kolb, Kayes ^20^ highlights both the strengths and criticisms of experiential learning, noting that while it offers a powerful model for professional education, it has been critiqued for its linearity and insufficient attention to cultural and social contexts. Nevertheless, the model’s emphasis on cycles of experience and reflection makes it particularly useful in environments where learners are expected to test, adapt, and refine new practices. Piaget’s developmental theory ^21^ also underpins this perspective, as it situates learning in processes of adaptation and accommodation, further reinforcing the idea that knowledge emerges from interaction with one’s environment.

Variation theory and phenomenography provide additional insights. Marton and Trigwell ^22^ argue that learning outcomes depend on the variation in how learners experience a phenomenon, while Marton ^23^ situates learning in the qualitatively different ways that people understand the same object of study. Applied to Living Labs, these perspectives suggest that participants’ diverse professional backgrounds and interpretations enrich the collective learning process, allowing new insights to emerge through dialogue and comparison.

Kolb and Kolb ^24^ further refine experiential learning by emphasizing that it should be seen not merely as an individual process but as part of broader experiential learning systems, integrating learners, facilitators, and environments into dynamic ecosystems. This view resonates strongly with the Living Lab context, where the interplay of actors, tools, and real-world settings produces opportunities for learning that extend beyond isolated experiences.

Taken together, experiential learning theories underscore the potential of Living Labs to serve as structured environments where professional practice is examined, questioned, and adapted through cycles of experience and reflection. They also highlight the need for facilitation and variation in design to ensure that different perspectives contribute to the learning process.

### Adult learning and andragogy

Theories of adult learning provide a complementary framework for understanding why Living Labs are particularly well suited to professional training. Knowles ^12, 13^ articulated the principles of andragogy, emphasizing that adults are self-directed, bring prior experience to learning, and are motivated when education is problem-centered and directly applicable to their professional lives. These assumptions resonate strongly with the design of Living Labs, where participants work with authentic challenges, contribute expertise, and co-create solutions that align with their operational contexts.

Further elaborations on adult learning highlight its complexity. Illeris ^25, 26^ argues that adult learning is distinctive because it integrates cognitive, emotional, and social dimensions. This holistic perspective underscores that professional education involves more than the acquisition of skills; it requires engagement with identity, motivation, and interaction with peers. Living Labs, with their dialogical and participatory structures, accommodate these dimensions by creating spaces where learners negotiate meaning, test assumptions, and reflect on experiences collectively.

Transformative learning theory extends this understanding by focusing on the potential for adults to shift their frames of reference. Mezirow ^14^ situates transformative learning in critical reflection, dialogue, and the confrontation of disorienting dilemmas. Within Living Labs, the introduction of unfamiliar tools, new data practices, or divergent professional perspectives can serve as catalysts for such reflection, encouraging participants to re-examine their assumptions and adapt their practices. Hoggan ^27^ further develops this by proposing analytical tools for identifying transformative outcomes, stressing the importance of both perspective change and practical application in assessing adult learning.

Together, these contributions show that Living Labs align with key adult learning theories by offering opportunities for self-directed, problem-centered, and transformative engagement. They allow professionals not only to acquire new knowledge but also to critically reflect on their roles and practices in ways that support both immediate application and long-term professional development.

### Synthesis: towards an integrated framework

The review of literature demonstrates that Living Labs embody features that resonate across several strands of educational theory. Experiential learning emphasizes cycles of experience, reflection, and experimentation ^10, 11, 20^, and Living Labs provide precisely the authentic contexts in which such cycles can unfold. Adult learning theories stress self-direction, problem-centeredness, and transformative reflection ^13, 14, 26, 27^, all of which are central to the design and facilitation of Living Lab activities. The perspective of Communities of Practice highlights the social and collaborative dimensions of learning ^15, 28^, which are inherent in the multi-actor interactions that characterize Living Labs.

Taken together, these perspectives position Living Labs as hybrid ecosystems of innovation and learning. They are not only infrastructures for testing and refining technologies but also pedagogical environments that allow professionals to engage in cycles of experiential learning, negotiate meaning collectively, and critically reflect on their practices. This synthesis underscores the value of Living Labs as settings where educational theory and innovation practice converge, offering a basis for capacity building in complex professional domains such as security and law enforcement.

These relationships are summarized in Table 1, which integrates the main educational frameworks with corresponding Living Lab features and pedagogical implications.

**Table 1.** Conceptual integration of Living Labs and adult learning frameworks.

Framework	Core principles	Living Lab features	Educational implications
Experiential learning (Kolb, ^10, 11^; Kayes, ^20^; Piaget/Wadsworth, ^21^; Marton & Trigwell, ^22^; Kolb & Kolb, ^24^)	*Learning occurs through cycles of experience, reflection, conceptualization, and experimentation*	*Authentic scenarios, iterative testing, feedback loops*	*Supports applied learning, reflection-in-action, and adaptation to operational contexts*
Adult learning (Knowles, ^12, 13^; Illeris, ^25, 26^; Mezirow, ^14^; Hoggan, ^27^)	*Self-direction, problem-centered learning, critical reflection, integration of cognitive–emotional–social dimensions*	*Participant agency, relevance to practice, disorienting dilemmas, dialogical exchanges*	*Promotes professional autonomy, transformative reflection, and holistic engagement*
Communities of Practice (Wenger, ^15^; Snyder & Wenger, ^29^; Campbell, ^28^)	*Learning emerges from participation, mutual engagement, and shared meaning-making*	*Multi-actor collaboration, co-creation of practices, peer dialogue*	*Fosters collective capacity building, identity development, and professional resilience*

This synthesis demonstrates that Living Labs can be conceptualized as intersections of multiple educational traditions. They integrate experiential processes, adult learning principles, and social collaboration, producing environments where learning is authentic, dialogical, and transformative. In this sense, Living Labs offer not only a methodological tool for innovation but also a conceptual framework for professional education in complex, rapidly evolving fields.

## Methodology

This study employed a qualitative research design in order to examine how Living Labs can serve as environments for professional learning within the framework of EU-funded security projects. A qualitative approach was chosen because it enables an in-depth exploration of meanings, experiences, and perspectives, rather than relying on pre-defined variables or quantitative measurement. As Cohen, Manion, and Morrison ^29^ argue, qualitative inquiry is particularly valuable when the aim is to understand complex social and educational processes from the viewpoint of those directly involved. This perspective aligns with the objectives of the present study, which sought to investigate how participants experienced the pedagogical dimensions of Living Labs in the Horizon Europe project TENACITy.

**Research design.** The study was structured around two complementary sources of evidence: semi-structured interviews with Passenger Information Unit officers and the analysis of project documentation. The combination of these sources allowed for methodological triangulation, thereby strengthening the validity of the findings ^30^. The interviews offered insights into participants’ lived experiences, capturing how they engaged with Living Lab activities and how they perceived learning outcomes. At the same time, project documents such as the TENACITy Grant Agreement and Deliverable D4.1 Training Methodology and Curricula ^31^ provided institutional context and clarified the intended role of Living Labs within the broader project framework. By integrating first-hand accounts with documentary sources, the research design allowed findings to be situated within both micro-level (individual experience) and macro-level (project objectives) perspectives.

**Data collection.** Data were collected through semi-structured interviews with officers who participated in the Living Lab activities. The choice of semi-structured interviews was deliberate: while structured enough to ensure consistency across participants, the format was flexible enough to allow officers to elaborate on their experiences and to highlight aspects they themselves considered important. Interviews focused on themes such as engagement, reflection, application of knowledge, and perceptions of support, thereby linking directly to the theoretical framework of experiential and adult learning. Importantly, no trainers or facilitators were included in the sample, as the aim was to prioritize the voices of end-users who were the primary intended beneficiaries of the Living Lab sessions. This focus reflects the principle of learner-centeredness in adult education ^13^, recognizing that the perspectives of practitioners are essential to evaluating the pedagogical value of innovative training formats.

**Reflexivity and positionality.** The researcher’s dual role as both a consortium member and an analyst was a defining feature of the study. On the one hand, being part of the project facilitated access to participants and provided a deep contextual understanding of the Living Lab activities. On the other hand, this insider position carried the risk of bias, as familiarity with the project could influence the interpretation of data. Reflexivity was therefore a central component of the research process. Following Lincoln and Guba’s ^30^ principle of trustworthiness, the researcher actively engaged in critical self-reflection, examining how their role might shape the questions asked, the interactions during interviews, and the analysis of responses. Steps taken to address these concerns included carefully designing interview guides to minimize leading questions, maintaining a clear separation between facilitation and research roles, and reviewing transcripts iteratively to ensure that interpretations were grounded in participants’ own words. This reflexive stance is consistent with Illeris’ ^26^ recognition that adult learning research must take into account not only cognitive dimensions but also the relational and contextual factors that influence both the research process and the learning process itself.

**Data analysis.** The interview transcripts were analyzed thematically, following the approach outlined by Braun and Clarke ^33^. Thematic analysis was chosen because it provides a flexible yet rigorous method for identifying, analyzing, and reporting patterns within qualitative data. The process began with familiarization with the transcripts, followed by generating initial codes to capture key features of the data. These codes were then organized into potential themes, which were refined iteratively by reviewing them against both the coded extracts and the entire dataset. This cyclical process ensured that the themes were rooted in the participants' accounts while also aligning with the theoretical frameworks guiding the study. NVivo software was utilized to support the organization and management of data, creating a structured audit trail of the coding process and enabling the researcher to work systematically across multiple transcripts. The use of NVivo complemented rather than replaced close interpretive engagement, ensuring that immersion in the data was maintained while also enhancing transparency and rigor in the analysis ^29^.

**Ethical considerations.** Given the professional context of the study, ethical issues were of particular importance. Participants were provided with clear information about the aims of the research, the voluntary nature of their participation and the ways in which their data would be used. Informed consent was obtained in writing prior to each interview. To protect confidentiality, all identifying information was removed from transcripts and participants are referred to only in general terms. No sensitive operational details were included in the analysis. The research was conducted within the ethical framework of the Horizon Europe TENACITy project, which complied with the EU General Data Protection Regulation (GDPR). While formal approval from a national ethics committee was not required, the procedures followed reflect established standards of ethical practice in qualitative educational research ^29, 30^.

**Trustworthiness.** The quality of qualitative research is often assessed through the criteria of credibility, dependability, confirmability, and transferability ^30^. In this study, credibility was strengthened through triangulation of interviews with project documentation, ensuring that participants’ accounts were contextualized within institutional frameworks. Dependability was supported by maintaining a transparent record of research procedures, from the development of interview guides to the coding process. Confirmability was enhanced through reflexivity, with the researcher critically examining their own role and potential biases throughout the study. Transferability, discussed in more detail below, was pursued by providing rich descriptions of the context and participants’ experiences, enabling readers to assess the applicability of the findings to other settings.

**Transferability**. As Larsson ^34^ argues, the value of qualitative research lies not in statistical generalization but in providing insights that are meaningful and relevant beyond the immediate case. This study does not claim universal applicability, but its findings may resonate with other professional training contexts where innovation and learning intersect. By offering thick descriptions of how officers engaged with Living Lab activities, the research provides a basis for readers to judge whether similar dynamics might be observed in their own contexts. Transferability is further supported by the use of established learning theories, including Kolb’s experiential cycle, Knowles’ andragogy, Mezirow’s transformative learning and Wenger’s communities of practice, which provide conceptual anchors that extend beyond the particularities of the TENACITy project. In this way, the study contributes both context-specific insights and broader conceptual reflections that may inform the design of training in other EU-funded projects.

## Results

The analysis of the interviews revealed five interrelated themes that captured the ways in which PIU officers engaged with the Living Lab environment and how these experiences contributed to their learning. These themes concern active engagement in Living Lab activities, processing and dialoguing, linking experience to theory and practice, testing and applying new knowledge, preparation, support, and learning conditions. Taken together, they illustrate both the opportunities and the constraints of using Living Labs as pedagogical environments in security-focused research projects.

The **first theme**, active engagement in Living Lab activities, reflected the officers’ emphasis on participation as a prerequisite for meaningful learning. Interviewees consistently highlighted that they were more motivated when actively involved in scenarios that simulated realistic operational conditions. Hands-on interaction with prototype tools allowed them to experience challenges that resonated with their professional routines, while also exposing them to functionalities and analytical processes that were unfamiliar. Engagement was not uniform, however, as the degree of involvement depended on the maturity of the tools and the clarity of the exercise design. Where tools were underdeveloped or datasets lacked authenticity, participants reported a sense of detachment that limited their ability to connect fully with the tasks.

The **second theme**, processing and dialoguing, underscored the importance of reflective exchanges during and after Living Lab sessions. Officers valued the opportunity to discuss their observations with peers and project partners, describing dialogue as essential for making sense of their experiences. Reflection was most effective when structured opportunities for feedback were provided, enabling participants to articulate both the practical relevance and the limitations of the tools under examination. This dialogical dimension extended beyond individual insights, as group discussions often generated new ideas for tool improvement and fostered a shared sense of ownership over the innovation process. Where dialogue was less systematically facilitated, however, officers noted that reflection remained superficial and fragmented.

The **third theme**, linking experience to theory and practice, captured the ways in which officers situated their Living Lab participation within broader professional and conceptual frameworks. Several interviewees reported that the activities helped them to connect their operational expertise with theoretical concepts such as data-driven risk assessment and cross-border cooperation. In this sense, the Living Lab served as a bridge between abstract knowledge and concrete tasks. Participants were able to recognize how specific analytical techniques might be integrated into existing workflows, while also critically evaluating whether the proposed solutions aligned with the legal and procedural requirements of PIUs. This linking process was facilitated when facilitators explicitly encouraged participants to situate their experiences within the wider policy and training context, but it was less effective in sessions that focused narrowly on technical demonstration.

The **fourth theme**, testing and applying new knowledge, related to the officers’ capacity to experiment with new tools and approaches. Participants described Living Labs as environments where they could take risks and explore alternative strategies without the operational pressures that characterize their daily work. This sense of safe experimentation allowed them to develop familiarity with advanced features, to test potential workflows, and to anticipate how new solutions might function in practice. Yet, the absence of fully operational datasets limited the transferability of insights. Officers emphasized that while synthetic data could serve an illustrative purpose, it often lacked the complexity and unpredictability of real passenger information, thereby reducing the validity of the lessons learned.

Finally, the **theme** of preparation, support, and learning conditions pointed to the structural factors that shaped participants’ experiences. Officers stressed that effective learning required adequate preparation, clear guidance, and supportive facilitation. When Living Lab sessions were well planned, with defined objectives and structured exercises, participants reported higher levels of engagement and more sustained learning outcomes. Conversely, insufficient preparation, lack of clarity, or technical malfunctions hindered their ability to focus on learning and reduced the perceived value of the activities. Several interviewees also highlighted the importance of continuous support, noting that learning in a Living Lab did not end with a single session but required follow-up opportunities to consolidate knowledge and apply insights in operational contexts.

Taken together, these findings suggest that Living Labs possess considerable potential to support experiential learning among PIU officers, but their effectiveness is contingent on the conditions under which they are designed and implemented. Engagement, reflection, integration with theory, experimentation, and structured support emerged as essential components for maximizing the pedagogical value of these environments. At the same time, challenges such as tool immaturity, dependence on synthetic data, and uneven facilitation highlight the need for intentional alignment between innovation and education when embedding Living Labs into research projects.

## Discussion

The analysis shows that Living Labs create conditions that align strongly with theories of experiential, adult, and social learning, while also exposing challenges that constrain their potential. The findings therefore illustrate both the opportunities and the limitations of using Living Labs as pedagogical infrastructures within EU-funded security research.

The theme of **active engagement and concrete experience** highlights the importance of authentic participation as a foundation for learning. Officers reported that hands-on interaction with prototype tools and scenarios provided valuable opportunities to connect training activities with their operational tasks. This resonates with Kolb’s ^10, 11^ emphasis on concrete experience as the entry point into cycles of learning, as well as with Piaget’s developmental theory of adaptation ^21^. Yet engagement was conditional: when the activities lacked realism due to immature tools or synthetic datasets, learners were unable to immerse themselves fully in the experience. Kayes ^20^ reminds us that experiential learning is not guaranteed by participation alone but requires structures that support depth and relevance. These findings confirm that the pedagogical value of Living Labs depends on the authenticity of the environment and the maturity of the resources provided.

The second theme, **processing and dialoguing**, emphasizes the role of reflection and collective sense-making. Structured opportunities for feedback and exchange allowed officers to articulate insights, critically assess the tools, and integrate multiple perspectives. This process reflects Mezirow’s ^14^ view that dialogue is central to transformative learning, enabling adults to reconsider assumptions and reframe their practices. It also aligns with Illeris’ ^26^ insistence that adult learning involves not only cognitive but also social and emotional dimensions, which are activated when participants negotiate meaning together. However, where facilitation was insufficient, dialogue remained superficial, limiting the depth of reflection. These findings suggest that Living Labs require intentional facilitation strategies to ensure that dialogue moves beyond description to critical engagement.

The third theme, **linking experience to theory and practice**, illustrates how Living Labs serve as bridges between abstract frameworks and operational application. Officers described how sessions helped them situate emerging technologies within the procedures of their Passenger Information Units, thereby translating innovation into professional relevance. This dynamic reflects Knowles’ ^13^ principle that adults learn best when education is problem-centered and directly applicable to their work. It also echoes Wenger’s ^15^ argument that learning emerges through participation in communities of practice, where new knowledge is tested and contextualized in shared professional settings. Yet this linking was not consistent across sessions. In the absence of explicit prompts or conceptual framing, participants tended to treat Living Labs as demonstrations of tools rather than as opportunities for integrated learning. These observations underscore the need for facilitators to actively scaffold the connection between experience, theory, and practice.

The fourth theme, **testing and applying new knowledge**, highlights how Living Labs provide participants with opportunities for safe experimentation. Officers valued the chance to trial new tools and explore alternative strategies in an environment free from the operational risks of real investigations. This corresponds to the stage of active experimentation in Kolb’s ^10, 11^ cycle and to Wenger’s ^15^ perspective that learning is grounded in practice. However, the reliance on synthetic datasets limited the authenticity of these experiments, reducing the extent to which insights could be transferred into operational contexts. Illeris ^25^ points out that learning requires the integration of both cognitive engagement and situational relevance; when realism is lacking, the capacity for genuine application is diminished. This tension underscores a recurring challenge in research-driven training: the need to balance ethical and legal constraints with the imperative of authenticity in professional learning.

The fifth theme, **preparation, support, and learning conditions**, demonstrates that Living Labs are highly sensitive to design and facilitation. Officers stressed that effective learning depended on clear objectives, structured exercises, and reliable technical conditions. In well-prepared sessions, participants reported greater engagement and more sustained learning outcomes. Conversely, poorly structured or technically unstable sessions hindered focus and reduced perceived value. These findings echo Illeris’ ^26^ holistic model, which highlights that adult learning depends both on the quality of the material and on motivation and environmental support. They also reflect Campbell’s ^28^ application of communities of practice to policing, which shows that effective collaborative learning requires organizational and structural conditions that enable trust, dialogue, and continuity.

**Limitations.** While the study provides valuable insights, several limitations should be acknowledged. The participant sample was relatively small and limited to officers from Passenger Information Units, which constrains the diversity of perspectives. The use of synthetic data, though necessary for ethical compliance, reduced the ecological validity of the learning environment. Furthermore, the researcher’s dual role as both participant and observer may have influenced interpretation, even though reflexivity was applied throughout the process. These limitations confirm Larsson’s ^34^ point that qualitative research produces knowledge that is context-bound and partial, requiring careful interpretation rather than claims of universal generalization.

**Transferability.** Despite these constraints, the findings have relevance beyond the immediate study. The features identified (authentic engagement, structured reflection, peer dialogue, experimentation, and supportive conditions)are not unique to aviation security but can be applied to other domains of law enforcement and professional training. Wenger’s ^15^ theory of communities of practice suggests that learning processes rooted in participation and shared meaning-making are broadly transferable across professional contexts. Campbell’s ^28^ findings in policing reinforce this view, showing that collaborative learning communities can strengthen adaptability in complex environments. In this sense, the study’s insights contribute to a wider discussion on how EU-funded projects can integrate Living Labs as sustainable infrastructures for professional education, supporting both technological innovation and capacity building.

Taken together, the expanded discussion demonstrates that Living Labs operationalize multiple dimensions of adult learning theory. They provide contexts for experiential cycles, spaces for critical reflection, opportunities for practice-based experimentation, and conditions for collaborative learning. At the same time, their effectiveness depends on authenticity, facilitation, and structural support. Recognizing both their potential and their limitations is essential if they are to be positioned as integral components of professional training within EU research frameworks.

## Policy and training implications

The findings of this study suggest that Living Labs should be viewed not only as instruments of innovation but also as infrastructures with considerable pedagogical value. This dual role carries implications for the way professional training is designed and implemented within the framework of EU-funded projects.

From an adult learning perspective, Living Labs demonstrate how professional education can be structured around the principles of self-direction, problem-centeredness, and immediate applicability ^13^. Officers participating in the Living Lab sessions were not passive recipients of training but active contributors, drawing on their expertise to shape the exercises. This reflects the need for training models that recognize learners as resources and co-creators of knowledge, rather than as audiences for instruction.

The findings also highlight the importance of holistic learning conditions, in line with Illeris’ ^26^ emphasis on the interplay of cognitive, emotional, and social dimensions. Participants’ reflections confirmed that effective learning required not only relevant tasks but also supportive facilitation, authentic contexts, and opportunities for dialogue. Policymakers and project designers should therefore ensure that training components embedded in research projects do not treat learning as an add-on but as an integral, intentionally designed process.

From the perspective of communities of practice, Living Labs show how learning emerges from participation and interaction ^15^. Officers engaged in shared reflection and peer dialogue, processes that contributed to collective meaning-making and professional identity formation. Campbell ^28^ demonstrates that such collaborative learning is particularly relevant in policing contexts, where adaptation to evolving challenges depends on networks of practice rather than isolated expertise. Embedding Living Labs in training design thus fosters not only individual learning but also organizational and community-level capacity building.

Finally, the study underscores the issue of sustainability. Living Labs are often tied to the lifespan of specific projects, yet their value lies in their potential to support ongoing professional learning. As Mezirow ^14^ argues, adult education is transformative when it enables learners to critically reflect on their frames of reference and adapt over time. Ensuring that Living Labs continue beyond project timelines would create opportunities for sustained cycles of reflection, experimentation, and adaptation, thereby reinforcing their contribution to both innovation and professional development.

In sum, the policy and training implications of this study emphasize that Living Labs should be intentionally integrated into the educational dimension of EU-funded projects. They represent infrastructures where innovation and learning reinforce one another, creating conditions for professionals to acquire, test, and critically reflect on knowledge in authentic contexts. Recognizing and sustaining this dual function would strengthen the capacity of law enforcement and other professional communities to meet the demands of rapidly changing environments.

## Conclusion

This study has examined the pedagogical role of Living Labs in the context of EU-funded security research, focusing on how they enable experiential and collaborative learning for officers in Passenger Information Units. Drawing on qualitative interviews and project documentation, the analysis identified five interrelated themes: active engagement, processing and dialoguing, linking experience to theory and practice, testing and applying new knowledge, and the influence of preparation and learning conditions. These dimensions illustrate both the potential and the limitations of Living Labs as environments for professional education.

The findings confirm that Living Labs align closely with established theories of adult learning. They operationalize Kolb’s ^10, 11^ experiential cycle by providing contexts for concrete experience, reflection, conceptual linking, and experimentation. They embody Knowles’ ^13^ principles of andragogy by situating learning in problem-centered, relevant, and self-directed contexts. They resonate with Illeris’ ^26^ view of adult learning as an integration of cognitive, emotional, and social dimensions, and with Mezirow’s ^14^ emphasis on critical reflection and perspective transformation. Finally, they reflect Wenger’s ^15^ notion of communities of practice, as participants learn not in isolation but through dialogue, participation, and shared meaning-making.

At the same time, the study underscores important constraints. Tool immaturity, reliance on synthetic datasets, and uneven facilitation limited the authenticity and depth of learning. These challenges highlight that the pedagogical value of Living Labs is not inherent but depends on intentional design, adequate preparation, and supportive conditions. As Illeris ^25^ and Kayes ^20^ remind us, adult learning is contingent on the quality of both the experience and its facilitation; without these, opportunities for reflection and transformation may remain underdeveloped.

The implications of these findings extend beyond the case of aviation security. The features identified in this study are relevant to other areas of law enforcement and professional training, where practitioners must adapt to rapidly evolving technological and organizational landscapes. As Wenger ^15^ and Campbell ^28^ show, collaborative learning rooted in communities of practice can enhance adaptability across diverse contexts. Embedding Living Labs more systematically into EU-funded projects can therefore strengthen the integration of innovation and training, ensuring that technological developments are accompanied by capacity building among end-users.

Future research could deepen these insights by exploring comparative cases across different domains, examining how variations in facilitation, data authenticity, and stakeholder composition influence learning outcomes. There is also a need to assess the long-term sustainability of Living Labs as infrastructures for professional education beyond project lifespans. Addressing these questions would contribute to a more comprehensive understanding of how Living Labs can be institutionalized as pedagogical spaces within European training ecosystems.

In conclusion, Living Labs represent hybrid infrastructures that merge innovation and learning. When designed and facilitated with pedagogical intent, they create environments where professionals engage authentically with new tools, reflect critically on their practices, and collectively build capacity to meet complex challenges. Recognizing and sustaining this dual role is essential if EU-funded research projects are to achieve their full potential in bridging technological advancement and professional development.

## Ethics and consent statement

This study involved qualitative interviews with law enforcement professionals. Participants were provided with an information sheet outlining the objectives, procedures and use of data in the research. Informed consent was obtained in writing prior to each interview and participation was entirely voluntary. All data were anonymised to ensure confidentiality. This study was conducted in accordance with the principles of the Declaration of Helsinki. Formal approval from a national ethics committee was not required, as the study was conducted within the framework of the Horizon Europe project TENACITy and complied with the ethical standards and data protection requirements set by the consortium, including the GDPR.

## Data Availability

The data underlying this study consist of anonymised interview transcripts with officers from Passenger Information Units. Due to the sensitive nature of the material and the potential risks to participants’ confidentiality, the transcripts cannot be made openly available. Summaries of the anonymised data that support the findings of this article are available upon reasonable request from the corresponding author ( k.margaros@kemea-research.gr). Access will be granted for research purposes only, subject to compliance with EU and institutional data protection regulations. Project documentation, including Deliverable D4.1 *Training Methodology and Curricula*, is publicly available through the European Commission’s CORDIS database: https://cordis.europa.eu/project/id/101074048/results. The TENACITy Grant Agreement (No. 101074048) is an internal document between the European Commission and the consortium and is therefore not publicly accessible. Zenodo: Living Labs as catalysts for experiential learning in law enforcement training: Insights from the TENACITy project ^35^. DOI: https://doi.org/10.5281/zenodo.17491498 Margaros, K., & Aposkiti, C. (2025) This project contains the following underlying data: Consent_Form Information sheet for participating in research Information sheet on personal data processing Questionnaire Data are available under the terms of the Creative Commons Attribution 4.0 International
