# Supplementary material for: Living Labs as catalysts for experiential learning in law enforcement training: Insights from the TENACITy project
Source: Open Res Eur. 2025 Dec 5;5:375. [Version 1] doi: 10.12688/openreseurope.21573.1 (PMC12828252; doi:10.12688/openreseurope.21573.1)
Supplement: Peer review history

# Reviewer report: Reviewer response for version 1

**DOI:** 10.21956/openreseurope.23340.r66880

**Published:** 2026-01-22

**Authors:** Heleen Vreugdenhil

**Affiliations:** 1 Delft University of Technology, Delft, The Netherlands

Theory: you provide a good overview of the relevant learning theories, but I suggest to have a look at learning pathways in Living Labs as developed by Bhatta et al., 2025. While the current conceptual lens confirms why and how LLs are useful for professional learning, with such an addition you could even better indicate the process of learning in LLs.

 Furthermore, you could make a better link between learning and innovation as the two are connected, and learning through experience, knowledge exchange and experiments is a process always taking place in LLs. So your argument would be stronger if you could indicate that learning could be enhanced e.g. by providing it specific attention in the design and operation of the LL. 

 You might want to reconsider adult learning into professional learning. With adult learning you could also refer to universities and higher educational schools, while your audience are professionals. 

 Research design & data collection: please extend these (e.g. who/how many people were interviewed and when, what type of questions were asked, what documents were used ? try to specify your triangulation approach, you could also consider using a mixed methods approach)

 Data analysis: you describe the process, but can you be more precise, e.g which themes did you elicit? Or can you provide overviews in supplementary material?

 Results and discussion are clear, however, to give more meaning to them and particularly when you discuss the relevance to law enforcement, the paper would benefit from quotes from interviewees or documents, to deepen the insights in learning and the generalizability. I really like your discussion on policy and training implications

If applicable, is the statistical analysis and its interpretation appropriate?

Not applicable

Is the study design appropriate and is the work technically sound?

Yes

Is the work clearly and accurately presented and does it engage with the current literature?

Partly

Are the conclusions drawn adequately supported by the results?

Yes

Are sufficient details of methods and analysis provided to allow replication by others?

No

Are all the source data and materials underlying the results available?

No

Reviewer Expertise:

Living Labs, innovation processes, public innovation, infrastructure

I confirm that I have read this submission and believe that I have an appropriate level of expertise to confirm that it is of an acceptable scientific standard, however I have significant reservations, as outlined above.

# Reviewer report: Reviewer response for version 1

**DOI:** 10.21956/openreseurope.23340.r66240

**Published:** 2026-01-21

**Authors:** Chris Croft

**Affiliations:** 1 University of Southern Mississippi, Hattiesburg, Mississippi, USA

Very good and applicable research study. I think the layout of experiential learning including literature review and connection to the topic is very solid. The various ELT themes are explained well and connected back to the topic in the paper to show the various levels from collected research. I think the manuscript add to ELT research and would be well-received by readers.

If applicable, is the statistical analysis and its interpretation appropriate?

Yes

Is the study design appropriate and is the work technically sound?

Yes

Is the work clearly and accurately presented and does it engage with the current literature?

Yes

Are the conclusions drawn adequately supported by the results?

Yes

Are sufficient details of methods and analysis provided to allow replication by others?

Yes

Are all the source data and materials underlying the results available?

Yes

Reviewer Expertise:

Experiential learning

I confirm that I have read this submission and believe that I have an appropriate level of expertise to confirm that it is of an acceptable scientific standard.

# Reviewer report: Reviewer response for version 1

**DOI:** 10.21956/openreseurope.23340.r66875

**Published:** 2026-01-13

**Authors:** Fumiya akasaka

**Affiliations:** 1 Research Institute on Human Societal Augmentation, National Institute of Advanced Industrial Science and Technology, Kashiwa,, Chiba, Japan

Thank you for giving me the valuable opportunity to review this article.

 This article discusses how living labs contribute to adult learning based on the EU-funded TENACITy project. Specifically, this study aims to analyze the pedagogical potential of living labs by drawing on theories of experiential learning, adult learning, and social learning. As the authors point out, while previous living lab studies have discussed the usefulness of living labs in learning contexts, their specific contributions have not been analyzed in detail. Considering social significance of Living Labs, their role as pedagogical infrastructure is a crucial point; thus, this study focuses on an important topic in living lab research.

 Through qualitative analysis of interview and document data obtained from the TENACITy project case, this study identified five interrelated dimensions regarding the contributions of living labs in learning contexts. Furthermore, based on these findings, this article presents implications for future policy development and training. The five dimensions and implications presented in this article include important insights from the lens of "living labs as pedagogical infrastructure" and are considered to expand existing knowledge in living lab studies.

 However, to make this article more valuable to readers, I would like to suggest the following three comments for the improvement of this article.

 1. The details on activities conducted within the TENACITY project

 This study analyzed a living lab designed within the TENACITY project to test data-driven tools for Passenger Information Units (PIUs) and to develop professional competences among participating officers. However, the current version of this article lacks detailed descriptions of the project's activities. I therefore recommend adding details on what activities to support and accelerate innovation, as well as activities to facilitate participants’ learning implemented within this living lab. This would enhance the credibility of the findings and discussions presented in the "Results" and "Discussion" sections in this article.

 2. Data obtained from interviews

 The “Results” section of this article presents the five dimensions regarding the living lab's contributions to the learning context. However, as this article does not present any examples of the interview data, which was used as the basis of this analysis, it remains unclear which specific statements (i.e., interview data) led to these findings. To enhance the transparency of this study, I recommend adding relevant key statement data for each of the five dimensions.

 3. Concrete Suggestions for the development and operation of living labs as pedagogical infrastructure

 The “Policy and training implications” section of this article describes some implications for utilizing living labs in learning contexts. However, it provides only abstract descriptions of the educational significance of living labs. Therefore, to enhance the practical contributions of this article, authors should provide more concrete and practical suggestions for practitioners who develop and operate living labs as pedagogical infrastructure. Such suggestions may include, for example, the key perspectives, considerations, and potential pitfalls for developing and operating living labs as pedagogical infrastructure. By doing so, this article would become more valuable not only academically but also practically.

If applicable, is the statistical analysis and its interpretation appropriate?

Not applicable

Is the study design appropriate and is the work technically sound?

Yes

Is the work clearly and accurately presented and does it engage with the current literature?

Yes

Are the conclusions drawn adequately supported by the results?

Yes

Are sufficient details of methods and analysis provided to allow replication by others?

Yes

Are all the source data and materials underlying the results available?

No

Reviewer Expertise:

Living labs, co-design, participatory design, service design, service engineering

I confirm that I have read this submission and believe that I have an appropriate level of expertise to confirm that it is of an acceptable scientific standard, however I have significant reservations, as outlined above.
